# Androgen signaling connects short isoform production to breakpoint formation at Ewing sarcoma breakpoint region 1

**DOI:** 10.1093/narcan/zcab033

**Published:** 2021-08-14

**Authors:** Taylor R Nicholas, Stephanie A Metcalf, Benjamin M Greulich, Peter C Hollenhorst

**Affiliations:** Department of Biology, Indiana University, Bloomington, IN 47405, USA; Medical Sciences, Indiana University School of Medicine, Bloomington, IN 47405, USA; Medical Sciences, Indiana University School of Medicine, Bloomington, IN 47405, USA; Medical Sciences, Indiana University School of Medicine, Bloomington, IN 47405, USA

## Abstract

Ewing sarcoma breakpoint region 1 (*EWSR1*) encodes a multifunctional protein that can cooperate with the transcription factor ERG to promote prostate cancer. The EWSR1 gene is also commonly involved in oncogenic gene rearrangements in Ewing sarcoma. Despite the cancer relevance of *EWSR1*, its regulation is poorly understood. Here we find that in prostate cancer, androgen signaling upregulates a 5′ *EWSR1* isoform by promoting usage of an intronic polyadenylation site. This isoform encodes a cytoplasmic protein that can strongly promote cell migration and clonogenic growth. Deletion of an Androgen Receptor (AR) binding site near the 5′ *EWSR1* polyadenylation site abolished androgen-dependent upregulation. This polyadenylation site is also near the Ewing sarcoma breakpoint hotspot, and androgen signaling promoted R-loop and breakpoint formation. RNase H overexpression reduced breakage and 5′ *EWSR1* isoform expression suggesting an R-loop dependent mechanism. These data suggest that androgen signaling can promote R-loops internal to the *EWSR1* gene leading to either early transcription termination, or breakpoint formation.

## INTRODUCTION

EWS, the protein encoded by Ewing sarcoma breakpoint region 1 (*EWSR1*), is ubiquitously expressed in humans and plays an essential role in normal development (1–3). In both Ewing sarcoma and certain leukemias, oncogenic gene rearrangements can fuse the 5′ end of *EWSR1* to the 3′ end of transcription factor encoding genes. One such fusion, *EWSR1/FLI1*, typifies Ewing sarcoma as it is found in 85% of cases (4). The fusion protein, EWS/FLI1, binds the genome through the C-terminal DNA binding domain of FLI1, while the N-terminal EWS portion functions as a strong transactivation domain (TAD) (5). Because of the outstanding recurrence of EWS/FLI1 in Ewing sarcoma, most of our understanding of EWS function comes from the fusion context. However, the wild-type EWS protein clearly has multivariate roles that are not well understood.

Both nuclear and cytoplasmic (6), EWS, the protein encoded by *EWSR1*, has transcription-dependent and transcription-independent roles. We have previously reported that EWS can act as a transcriptional co-activator in prostate cancer (7); other reported nuclear functions of EWS include the regulation of splicing and DNA damage repair (8–10). Sedimentation studies suggest that cytoplasmic EWS associates with dense, ribosome-containing fractions as well as lighter fractions that also contain the plasma membrane (11). Additionally, knockout studies have implicated *EWSR1* in meiosis, with null mice defective in spermatogenesis and oogenesis (1).

EWS belongs to a small family of proteins with conserved structure and partially overlapping functions, termed the FET (FUS, EWS, TAF15) family. FET family members have an N-terminal prion-like domain (PrLD) consisting of degenerate SYGQ repeats. This ‘low complexity domain’ has important transcription-related oncogenic properties as the N-terminus of all three FET proteins are found fused in various cancers to transcription factor genes (4,12,13). In the C-terminus, FET proteins bind nucleic acids with an RNA recognition motif (RRM) and a zinc finger domain (ZnF), punctuated by arginine-glycine-glycine rich (RGG) regions. At the very C-terminal end is a nuclear localization signal. Aside from cancer, all three FET family members are mutated in amyotrophic lateral sclerosis (ALS) and in frontotemporal dementia (FTD) (14,15). FET proteins have been demonstrated to form higher order structures that cause liquid demixing and formation of membraneless organelles through aggregation in the PrLD (16–18) or RNA binding (19). When reversible, these ‘droplets’ facilitate normal cellular processes such as cytoplasmic stress granule formation but when irreversible may form pathological plaques in disease (20).

Androgen receptor (AR) is a ligand-inducible nuclear hormone receptor that is important in development and disease. Once activated by androgens, AR translocates to the nucleus and functions as a transcription factor, controlling genetic programs important for growth and tissue determination (21). AR promotes male-specific characteristics and is involved in prostate cancer initiation and progression (22,23). In addition to transcriptional function, AR also promotes the chromosomal rearrangement responsible for the *TMPRSS2/ERG* gene fusion found in 50% of prostate tumors (24,25). Interestingly, like *EWSR1* null mice, *AR* null mice are defective in spermatogenesis from meiotic arrest (26), suggesting that *AR* and *EWSR1* can regulate similar phenotypes.

In this study, we find that androgen signaling regulates the *EWSR1* gene to produce different genetic outcomes important for cancer biology. In prostate cancer, we found that AR drives formation of a shortened *EWSR1* isoform that promotes cancer-associated phenotypes. We then used prostate cancer cells as a model to show androgen driven formation of a break in *EWSR1* at the same breakpoint hotspot that creates the *EWSR1/FLI1* oncogene in Ewing sarcoma. While it is known that AR promotes gene fusion formation in prostate cancer, an androgen-dependent mechanism for *EWSR1* breakage has not been shown. This is the first study to characterize direct androgen regulation of the *EWSR1* gene.

## MATERIALS AND METHODS

### RNA extraction and quantitative reverse transcription PCR

RNA was extracted using the RNAeasy kit (Qiagen) and DNase treated with the RNase-Free DNase kit (Qiagen) following manufacturer's protocols. 1 μg of total RNA was reverse transcribed using isoform specific primers (Supplementary Tables S1 and S2). RNA was measured by qRTPCR using standard curves as previously described (27). Expression was normalized to 18S and reported as three biological replicates each represented by the average of two technical replicates.

### Cell lines

VCaP, LNCaP and PC3 cells were obtained from ATCC. All cell lines were authenticated by the Oregon Health and Science University DNA Services Core and meet the > 80% STR match threshold (28). All lines were cultured as according to manufacturer guidelines. In androgen depletion experiments, VCaPs and LNCaPs were androgen starved using phenol red free media supplemented with charcoal stripped FBS (Gibco) for 48 h prior to R1881 (Sigma) exposure.

### Immunoblots

Whole cell extracts derived from equivalent cell numbers were electrophoresed on 10% SDS-PAGE, transferred to nitrocellulose membrane and blocked with 5% non-fat milk in TBST. Primary antibodies were applied in blocking buffer at manufacturer recommended dilution. Antibodies were tubulin (Sigma Aldrich T9026), HA (Sigma Aldrich H3663), AR (Abcam ab108341), GAPDH (Santa Cruz 0411 sc-47724), EWS (Santa Cruz G5 sc-28327), H3 (Cell Signaling D1H2) or ntEWS (Custom, Life Technologies). Secondary antibodies (anti-Rabbit or anti-Mouse HRP, Cytiva) were diluted to 1:12,500 in blocking buffer and, after washing, membranes developed with ECL (Pierce, Thermofisher).

### Viral transductions and transient transfections

Overexpression constructs were expressed in PC3s by retrovirus and have N-terminal 2xHA tags. Full length EWS was described previously (7), the N-terminal EWS isoform was cloned from LNCaP total RNA. The C-terminal EWS isoform and EWS (1–355aa) were cloned from full length using traditional PCR methods and ligation dependent cloning. AR was cloned from pCMV-FLAG-hAR (29) (a gift from Elizabeth Wilson; Addgene plasmid #89080; http://net.net/addgene89080;  RRID:Addgene_89080). See Supplementary Table S3 for cloning primers. The RNase H expression construct (ppyCAG_RNaseH1_WT (30) was a gift from Xiang-Dong Fu; Addgene plasmid #111906; http://n2t.net/addgene:111906; RRID Addgene_111906) was expressed in VCaP cells via transient transfection using TransIT 20–20 (Mirus). Cells were split two days post transfection for downstream experiments.

### Generation of CRISPR cells

The lentiCRISPR v2 plasmid was obtained from Addgene (a gift from Feng Zhang; Addgene plasmid #52961). gRNAs were designed and cloned into lentiCRISPR v2 according to the protocol provided by Addgene. The gRNA targeting upsteam the FOXA1:AR site was generated by annealing the 5′ primer caccgATCCGGGAGAAGTGATCTGTT and the 3′ primer aaacaacagatcacttctcccggatc (Supplementary Table S3). The gRNA targeting downstream the FOXA1:AR site was generated by annealing the 5′ primer caccgagctttgtagcattcttaccc and the 3′ primer aaacgggtaagaatgctacaaagctc. LentiCRISPR v2 plasmids containing gRNAs were packaged into lentivirus and exposed to VCaP cells in two rounds. CRISPR cells were used polyclonally, due to difficulty selecting clones in the VCaP line.

### Clonogenic growth, cell migration, and MTT assays

Transwell migration assays were preformed as previously described (27). Briefly, PC3s were plated into transwells (8 μM pore size, BD Bioscience) at a density of 500 000 cells/well in serum free media and allowed 48 h to migrate towards serum containing media. Migrated cells were then fixed, stained, and quantified; values are the mean and SEM of three biological replicates with two technical replicates each. Clonogenic growth was performed as previously described (7). 1000 PC3 or 5000 VCaP cells were seeded into a well of a six-well plate and allowed to grow for 10 days. VCaP cells were plated with VCaP-conditioned media. Colonies were fixed with 10% formalin, stained with 0.5% crystal violet (Sigma) in 25% methanol, and counted using Genesys software (Syngene). Reported colonies are the mean of three biological replicates with two technical replicates each. Cell proliferation was measured using the MTT (Calbiochem) assay as previously described (31). Briefly, 800 PC3 cells were seeded in a 96-well plate. Readings were taken 24 h after plating with time points continuing for 4 days. MTT reagent (5mg/ml in PBS) was added to cells and incubated for 4 h after which the MTT containing media was removed and DMSO added. After gentle agitation, absorbance (600nm) was measured using a microplate reader (ELx8200, BioTek Instruments). Cell proliferation was reported as three biological replicates each with five technical replicates.

### Precipitation assay

Protein precipitation via b-isox was performed as shown previously (32). Briefly, cell lysates were divided into four tubes and treated with 0, 10, 30 or 100 μM b-isox (Sigma). Lysates were incubated at 4°C with rotation for 1 h. Protein was precipitated by centrifugation and washed twice and protein aggregation was monitored by immunoblot.

### Chromatin immunoprecipitation (ChIP)

ChIP of indicated proteins was previously described (33) but after 2 days of androgen depletion and subsequent treatment with 10 nM R1881 or DMSO. Antibodies used for immunoblot were also used for ChIP. Briefly, crosslinking was carried out using 1% formaldehyde and quenched with glycine. Cells were lysed with ChIP cell lysis buffer (50 mM HEPES–KOH pH 8, 1 mM EDTA, 0.5 mM EGTA, 140 mM NaCl, 10% glycerol, 0.5% NP-40, 0.25% Triton X-100) supplemented with protease inhibitors (Sigma) and nuclei were isolated and washed with ChIP wash buffer (10 mM Tris–HCl pH 8, 1 mM EDTA, 0.5 mM EGTA, 200 mM NaCl) supplemented with protease inhibitors (Sigma). Nuclei were then sonicated using a Biorupter Pico for 30 s on, 30 s off for four rounds. Nuclear extract was added to dynabead-antibody conjugates and rotated for 4 h at 4°. Bead complexes were washed with IP wash buffer (20 mM Tris pH 7.9, 0.25% NP-40, 0.05% SDS, 2 mM EDTA, 250 mM NaCl) four times. Protein and RNA were degraded by Proteinase K (Sigma) and RNase A (5prime), respectively. DNA was purified by phenol chloroform extraction and QiaQuick PCR Purification Kit (Qiagen).

### ChIP-seq analysis

Raw ChIP-seq fastq files for AR from patient tumors and matched adjacent normal tissue (GSE70079), for TFs in VCaPs (GSE56086), and for TFs in LNCaPs (GSE83860) were downloaded from the SRA using SRA toolkit (http://ncbi.github.io/sra-tools/). When applicable, sequencing replicates and biological replicates were concatenated. Reads were aligned using Bowtie2 (34) and peaks were called using the default settings with MACS2 (35) after removing PCR duplicates with samtools (36). Motif analysis was performed with the AR motif (http://homer.ucsd.edu/homer/motif/HomerMotifDB/homerResults/motif6.info.html), AR half-site (http://homer.ucsd.edu/homer/motif/HomerMotifDB/homerResults/motif7.info.html), and FOXA1:AR motif (http://homer.ucsd.edu/homer/motif/HomerMotifDB/homerResults/motif5.info.html) using the FIMO tool on MEME-suite (37).

### Dot Blot

Nitrocellulose membrane was soaked in 6× SSC buffer (diluted in ddH_2_O from 20×; 3 M NaCl, 300mM trisodium citrate, pH 7) for 30 min to 1 h then assembled in a dot blot apparatus (BioRad). After washing with 100 μl TE buffer, 2 μg and 1 μg of digested genomic DNA in 100 μl of TE buffer was added to appropriate wells and vacuum filtered. Wells were washed twice with 2× SSC buffer (diluted in ddH_2_O from 20×). The membrane was washed for 30 s with 2× SSC buffer, air dried for 30 min, crosslinked using 0.12 J/m^2^ UV, then immunoblotted with S9.6 antibody (Abcam) at 1:1000 dilution.

### DRIP-qPCR

DRIP-qPCR was done as previously described (38) with the following changes. Genomic DNA was extracted from ∼3 million VCaP cells, immunoprecipitated with 8 μg of S9.6 antibody and 100 μl anti-mouse Dynabeads (Invitrogen), and DNA cleaned up using AMPure beads (Beckman Coulter Inc.). Enrichment is reported as percent IP/Input.

### Break-apart FISH

The *EWSR1* break-apart FISH probes were made by Lecia Biosystems. Break-apart FISH was performed as previously described (39). Breifly, 10 μl of probe was added to coverslips, sealed to slides with rubber cement, and incubated at 80C for 5 min. Coverslips were then placed in a humidification chamber at 37°C overnight. The following day, rubber cement was removed and coverslips washed as following: 2 min at RT with agitation with Wash Buffer 2 (2× SSC/0.1% NP-40), 2 min at 72°C without agitation with Wash Buffer 1 (0.4× SSC/0.3% NP-40), and 2 min at RT without agitation with Wash Buffer 2. Samples were then dehydrated with the following series of ethanol incubations: 70% EtOH for 1 min, 85% EtOH for 1 min, 100% EtOH for 1 min. After air drying, coverslips were mounted to slides and stained with DAPI. *EWSR1* is on chromosome 22; VCaP cells have three copies of this chromosome, thus signal from all alleles per nuclei was required for scoring. Breaks were scored by split green-red signal of at least one signal diameter as shown previously (39). Imaging was performed using a Nikon NiE using a 60× objective.

## RESULTS

### Androgen signaling upregulates an intronic polyadenylated *EWSR1* isoform

**Figure 1.**
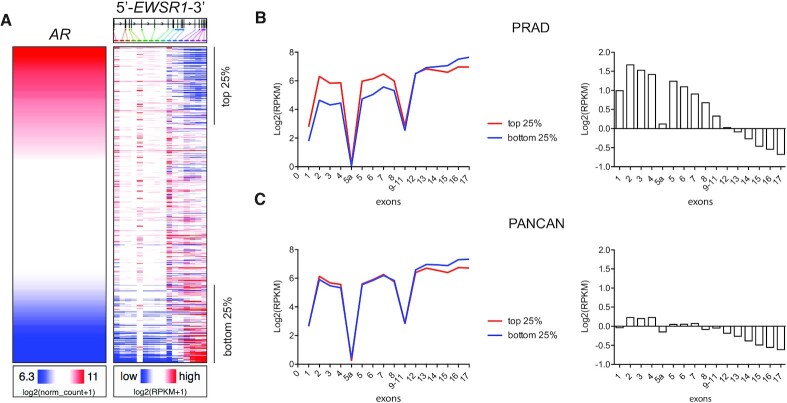
Androgen receptor expression correlates with high expression of 5′ EWSR1 exons. (**A**) *EWSR1* exon expression ranked by *AR* gene expression in primary prostate cancer patients (PRAD). (**B**) (left) Average expression of *EWSR1* exons in the samples with the highest 25% of *AR* expression (red) and with the lowest 25% of *AR* expression (blue) for the PRAD data set. (right) Difference in expression of the top 25% expression bin and the bottom 25% expression bin. (**C**) Same as in (B) but for the PANCAN TCGA data set. See also Supplementary Figure S1.

We previously found that EWS plays an important role in prostate cancer (7), therefore we investigated the relationship between *EWSR1* expression and *AR* expression, as AR is essential for prostate cancer development and progression. Using the prostate adenocarcinoma (PRAD) TCGA dataset housed on UCSC Xena Browser (https://xenabrowser.net (40), *EWSR1* mRNA level was ranked by *AR* gene expression from high to low in 550 patient tumor samples. Mean expression of all *EWSR1* exons was negatively correlated with *AR* levels (Supplementary Figure S1A). However, exon-level analysis found that the 5′ *EWSR1* exons were positively correlated with *AR* while the 3′ exons were inversely correlated (Figure 1A). Xena Browser by default normalizes the log_2_(RPKM + 1) values for each exon. To determine absolute expression levels, patient samples were separated into two bins: the top 25% of samples (high AR) and the bottom 25% of samples (low AR) and mean expression of each exon was plotted (Figure 1B, left). The 5′ *EWSR1* exons showed the greatest separation of signal between the two bins, with the exceptions of exon 1 (a 5′ UTR exon), exon 5a (a brain specific exon), and exons 9–11 (which Xena browser called as a single exon including the intervening introns). Exons 12 and 13 remained at a similar expression level, regardless of AR expression, while the 3′ most exons were expressed at lower levels when AR was high. Plotting the difference of the two bins (Figure 1B, right) reveals a pattern of progressively decreased exon expression across the *EWSR1* gene when AR levels are high. To understand the specificity of this observation, we performed the same analysis across multiple cancer types using the PANCAN TCGA data set (Figure 1C). In this case, only a modest (log_2_(RPKM) = ∼0.2) difference in 5′ exon expression between the two bins was seen, however the decrease in 3′ exons was still observed. Analysis of published ChIP-seq data (41) indicates higher RNA Polymerase II occupancy at the 5′ end of the *EWSR1* gene in the presence of the synthetic androgen R1881 (Supplementary Figure S1B). Together, these data indicate that AR signaling correlates with higher expression of 5′ *EWSR1* exons and suggests that AR might promote early termination of the *EWSR1* transcript.

**Figure 2.**
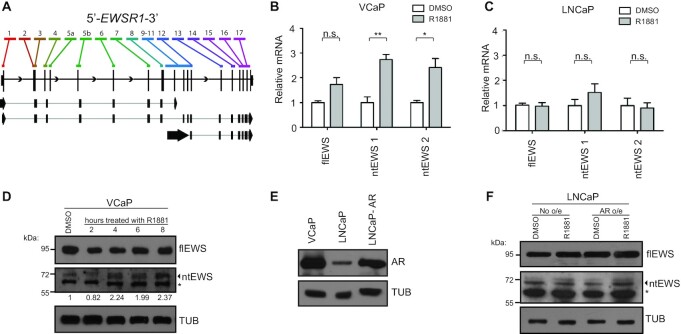
Androgen signaling upregulates an intronic polyadenylated EWSR1 isoform. (**A**) UCSC Xena Browser called exons for EWSR1 (top) with gene schematic (middle) and hg19 annotated EWSR1 isoforms (bottom). Arrows indicate 5′ and 3′ UTRs. (**B**, **C**) Normalized gene expression by isoform-specific qRTPCR for flEWS and ntEWS measured by one or two isoform-specific primer sets, respectively, in VCaP (B) and LNCaP (C) cells. Expression is normalized to 18S and relative to the DMSO condition. Shown is mean ± SEM for three replicates. All *P* values (*P < 0.05, ** *P* < 0.01) were obtained by t tests. (**D**) Immunoblot of ntEWS in VCaP treated with 10 nM R1881 for indicated time. Quantification of ntEWS bands are normalized to tubulin (TUB) and relative to DMSO. (**E**) AR immunoblot in indicated cell lines. (**F**) Immunoblot of flEWS, ntEWS, and TUB in LNCaP cells with or without AR overexpression treated with 10 nM R1881 for 4 h. Bands labeled with * indicate non-specific species. See also Supplementary Figure S2.

The differing expression levels of the 5′ and 3′ *EWSR1* exons suggests that *EWSR1* might not be transcribed as a single unit and that multiple *EWSR1* isoforms exist. In fact, the hg19 UCSC gene annotation (42) for *EWSR1* shows several isoforms (Figure 2A), including one (NM_001163287) that is composed of 5′ exons and terminates via an intronic polyadenylation event that generates an alternative last exon (ALE), exon 9 and 3′ UTR. Because this isoform encodes the N-terminus of the EWS protein, we have termed it the N-terminal isoform or ntEWS. Although annotated, *ntEWS* regulation and function has not been reported in the literature to our knowledge. Polya_db (version 3.2, http://exon.njms.rutgers.edu/polya_db/v3/), a database of polyA sites (PAS), captures the intronic polyadenylation event that forms *ntEWS* in 69.2% of samples (Supplementary Figure S2A and B). To investigate tissue specificity of *ntEWS*, we analyzed previously reported genomic 3′-sequencing data (43) and found that ntEWS is preferentially expressed in the testis (Supplementary Figure S2C), consistent with a male-specific and possibly androgen-regulated role.

Since all exons in *ntEWS* track with AR levels in patient tumors (besides exon 1 and 9, mentioned above), we tested whether androgen signaling would increase *ntEWS* RNA levels. Androgen responsive prostate cancer cell lines VCaP and LNCaP were treated with 10 nM of synthetic androgen R1881 for 24 h and changes in RNA was measured by isoform-specific qRTPCR. Primers detecting *ntEWS* span the novel exon-exon junction created by the intronic polyadenylation event and the *flEWS* specific primer set uses the first exon not present in *ntEWS*. Prostate specific antigen (PSA) RNA expression was used to verify androgen response (Supplementary Figure S2D). Treatment of VCaP cells with R1881 caused a significant upregulation in *ntEWS* mRNA level measured by two isoform-specific primer sets. Full-length *EWS* expression increased modestly but not to a significant level (Figure 2B). In contrast, treatment with R1881 did not increase the level of *ntEWS* in LNCaP cells (Figure 2C).

To measure levels of the ntEWS protein, we generated a polyclonal rabbit antibody (ThermoFisher) using the sequence encoded by the ALE as an epitope. It is important to note that this antibody is not affinity purified and is used in rabbit serum. Since other antibodies are present in rabbit serum, we needed to validate the ability to detect ntEWS using this reagent. The ∼70 kDa band was verified as ntEWS by the presence of a similar migrating band in lysates of ntEWS overexpressing PC3 cells and the absence of this band in lysates of empty vector expressing PC3 cells (Supplementary Figure S2E). Further, diminished levels of this band were observed using three independent shRNAs targeting ntEWS (Supplementary Figure S2F). To determine if ntEWS protein increased after androgen exposure, VCaP cells were treated for 2, 4, 6 or 8 h with R1881 and ntEWS protein levels were compared to that in vehicle treated cells (Figure 2D). After 4 h of R1881, ntEWS protein was increased roughly two-fold, corroborating the increase in *ntEWS* RNA seen in VCaP cells in Figure 2B.

We questioned whether the ability of R1881 to increase *ntEWS* levels in VCaP, but not LNCaP was due to differences in AR expression levels. In fact, AR is more abundant in VCaP cells than in LNCaP cells (Figure 2E), which corresponds to heightened AR activity measured by PSA expression (Supplementary Figure S2D). To test this idea, AR was transiently overexpressed in LNCaP cells to achieve expression levels similar to that in VCaP cells (Figure 2E) and ntEWS protein and PSA RNA were measured after treatment with R1881. AR overexpression in LNCaP cells allowed R1881 to promote a further increase in PSA (Supplementary Figure S2D), and, strikingly, mediated an R1881-dependent upregulation of ntEWS protein (Figure 2F).

### AR binding to Intron 5 of *EWSR1* directly regulates ntEWS expression

**Figure 3.**
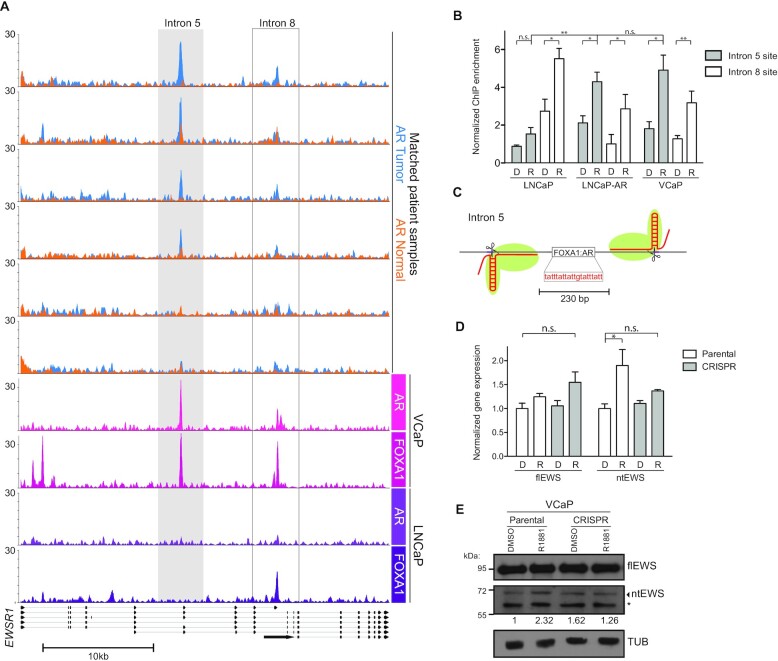
AR binding to Intron 5 of *EWSR1* directly regulates ntEWS expression. (**A**) Gene tracks for ChIP-seq of AR or FOXA1 in samples as labeled. Y-axis is log-transformed p-value. (**B**) ChIP-qPCR of AR in LNCaP and VCaP cells treated with DMSO (D) or R1881 (R) at the Intron 5 (gray) and Intron 8 (white) *EWSR1* sites. ChIP enrichment is normalized to a negative control region (*XKRT*) and the mean ± SEM for three replicates is shown. *P* values (*P < 0.05, ** *P* < 0.01) were obtained by *t* tests. (**C**) Depiction of the pgRNA targeting strategy for the FOXA1:AR binding site in intron 5 of *EWSR1*. (**D**) mRNA level of full length EWS (flEWS) or N-terminal EWS (ntEWS) in indicated cell lines treated with DMSO (D) or 10 nM R1881 (R) for 4 h, normalized as in Figure 2B. (**E**) Immunoblot of flEWS, ntEWS or tubulin (TUB) protein in parental or CRISPR VCaP cells treated as in (D), quantification as in Figure 2D. Bands labeled with * indicate non-specific species. See also Supplementary Figure S3.

To determine if AR regulation of the *EWSR1* gene is through direct binding, we analyzed published AR ChIP-seq datasets from patient tumor samples and matched adjacent normal tissue (44). At the *EWSR1* gene, AR is bound to two intragenic sites; one in intron 5, and one in the exon-intron boundary of exon 9 and intron 8 that overlaps with the PAS that produces ntEWS (Figure 3A). AR occupancy at the intron 5 site was particularly strong in 4/6 tumors and was higher than any matched normal peak, suggesting tumor-specific binding of AR to this site. These patient samples also exhibited high tumor:normal AR enrichment at the widely studied AREs near *KLK2* and *KLK3* suggesting higher overall AR binding (Supplementary Figure S3A).

AR ChIP-seq in VCaP cells treated with 10 nM R1881 for 2 h (45) shows similar localization observed in patient tumors at the *EWSR1* gene: modest signal at the intron 8 and robust signal at the upstream intron 5 site (Figure 3A). However, in LNCaP cells treated with 100 nM dihydrotestosterone (DHT) for 2 h (46), AR shows very low levels of binding to both sites (Figure 3A), suggesting that high expression level of AR is required to saturate these binding sites. The AR co-factor FOXA1 was found bound to both sites in VCaP cells and to the intron 8 site only in LNCaP cells. To reconcile treatment differences and verify binding, we performed ChIP-qPCR of AR in VCaP and LNCaP cells treated with 10 nM R1881 for 2 h. AR was significantly enriched at both sites when stimulated with R1881 in VCaP cells in a manner consistent with the ChIP-seq data (Figure 3B). In LNCaP cells, AR only showed significant binding to the intron 8 site (Figure 3B). To test if AR binding to the intron 5 site requires higher AR levels than what is found in LNCaP cells, ChIP was also performed in LNCaP cells transiently over-expressing AR. In these LNCaP-AR cells, AR occupied the intron 5 site to a similar extent as in VCaP cells (Figure 3B).

The sequence at each AR binding site identified in the ChIP-seq data was analyzed to identify androgen response elements (AREs). Three different AR position weight matrices (JASPAR) were run through the FIMO tool from MEME-suite (37). The most significant AR motif was a FOXA1:AR site in intron 5 (*P* = 5.06e–05). Since AR binding at the intron 5 site is more robust in tumors and VCaP cells, we focused on understanding the importance of AR binding to that site by a CRISPR-Cas9 disruption strategy. To avoid the limitations of targeting a short A:T rich sequence, paired guide RNAs (pgRNA) were targeted to flanking sequences (Figure 3C) as pgRNA targeting is an effective method to achieve targeted deletions (47–49). Cells with Cas9 targeted to the intron 5 ARE failed to upregulate ntEWS upon R1881 treatment while not affecting flEWS expression at both the mRNA (Figure 3D) and protein (Figure 3E) level. This suggests that the ARE in intron 5 is an important and specific *cis*-regulatory element for *ntEWS*.

### ntEWS promotes phenotypes related to oncogenesis

**Figure 4.**
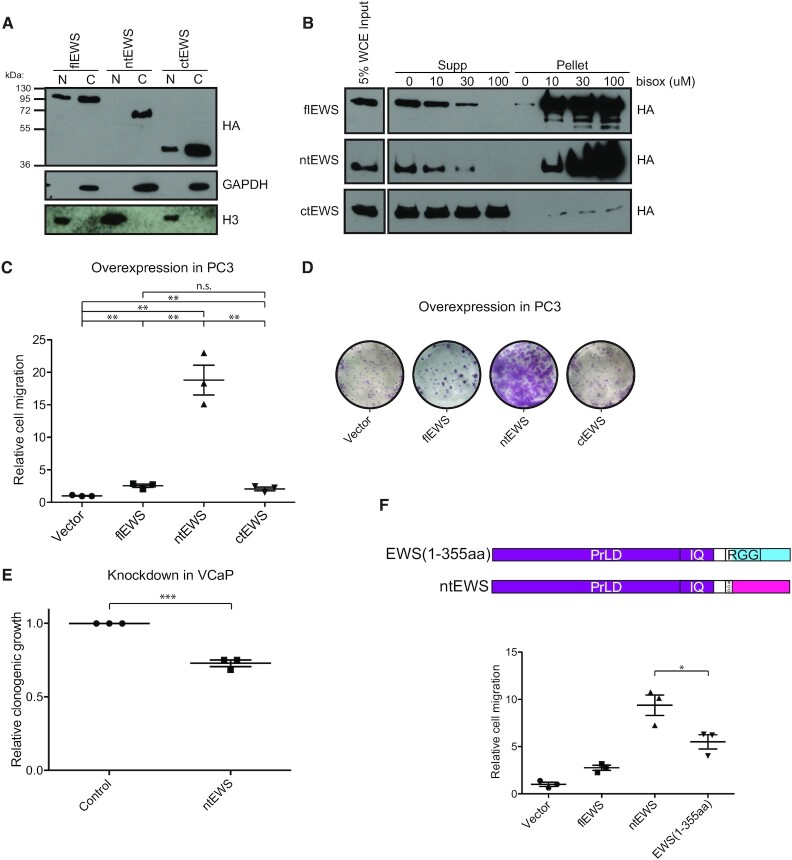
ntEWS promotes phenotypes related to oncogenesis. (**A**) Nuclear-cytoplasmic fractionation of 3xHA-flEWS, 3xHA-ntEWS and 3xHA-ctEWS in PC3 cells visualized by HA immunoblot. Nuclear and cytoplasmic fraction controls are H3 and GAPDH immunoblot, respectively. (**B**) B-isox precipitation assay of PC3 with 3xHA-flEWS, 3xHA-ntEWS, or 3xHA-ctEWS immunoblotted for HA. (**C**) Cell migration of EWS isoform overexpressing PC3 cells. Cell migration relative to the vector expressing cells is the mean ± SEM for three biological replicates with two technical replicates each. (**D**) Representative images of clonogenic growth of EWS isoform overexpressing PC3 cells. (**E**) Clonogenic growth of VCaP cells with ntEWS specific shRNA knockdown compared to shRNA targeting luciferase as a negative control. Mean ± SEM for three biological replicates. (**F**) Schematic diagram (top) of EWS 1–355 and ntEWS. Prion-like domain (PrLD), calmodulin binding domain (IQ) and arginine rich regions (RGG) are shown. ALE is colored pink. Cell migration (bottom) of PC3 cells expressing indicated constructs is shown relative to vector as the mean ± SEM for three biological replicates. All P values (***P < 0.001), **P < 0.01, *P < 0.05) were obtained by *t* test. See also Supplementary Figure S4.

Since the function of ntEWS has not been described in the literature, we cloned and overexpressed ntEWS in the androgen insensitive cell line PC3. For comparison, we also overexpressed flEWS and a predicted C-terminal isoform (ctEWS; NM_001163286; Figure 1A bottom) that shares no sequence homology to ntEWS (Supplementary Figure S4A). Nuclear-cytoplasmic fractionation of the over expressing lines showed the presence of both flEWS and ctEWS in the nucleus and cytoplasm; however, ntEWS was found exclusively in the cytoplasm (Figure 4A), consistent with previous reports of the nuclear localization signal in the C-terminus of EWS (50).

The N-terminal PrLD of EWS has been shown to form higher order structures seeded by various substrates in the context of flEWS and EWS/FLI1 (32,51). To determine the ability of ntEWS to form such structures, we performed a biotinylated isoxazole (b-isox) precipitation assay. Addition of the compound b-isox to cell lysates seeds precipitates formed by low-complexity domains like those found in the PrLD of EWS (51). We added increasing amounts of b-isox to PC3 cell lysates containing flEWS, ntEWS or ctEWS. Consistent with previous reports (51), flEWS was found in the b-isox seeded pellet in a concentration dependent manner. ntEWS was also pelleted by b-isox in a concentration dependent manner, while ctEWS, which lacks the PrLD, was not precipitated by b-isox, even at high concentrations (Figure 4B).

We next sought to determine the cellular impact of EWS isoform expression. The role of ntEWS in transwell cell migration and clonogenic growth of PC3 cells were examined, since flEWS can also regulate these phenotypes (7). Expression of flEWS induced significant cell migration and clonogenic growth compared to vector expressing PC3 cells, consistent with our previous work (7). Additionally, ctEWS expression promoted these phenotypes to a similar extent as flEWS. Strikingly, ntEWS expression in PC3 cells promoted dramatic increases in cell migration and clonogenic growth (Figure 4C and D). ntEWS expressing cells did not show increased proliferation compared to the flEWS and ctEWS expressing cells, measured by MTT assay (Supplementary Figure S4B), suggesting that ntEWS driven phenotypes are not a function of increased proliferation of these cells. To test if endogenous ntEWS promotes these phenotypes, ntEWS was knocked down in VCaP cells using shRNA 1 as shown in Supplementary Figure S2F. Knockdown of ntEWS, but not a control shRNA, significantly decreased VCaP clonogenic growth (Figure 4E and Supplementary Figure S4C).

The ntEWS protein includes amino acids encoded by the alternative last exon, which are not present in full-length EWS. To test if this region is important for function, or if the increased function of ntEWS is due only to truncation compared to full-length, we compared PC3 cells expressing HA-tagged ntEWS or EWS (1–355aa), which is the same length, but includes the amino acids encoded by full-length *EWSR1* (Supplementary Figure S4D). Interestingly, EWS (1–355aa) induced cell migration, 6-fold more than the vector expressing cells, however not to the same extent as ntEWS, which drove cell migration 10-fold more than the control (Figure 4F). This suggests that the PrLD, which is present in both proteins, can promote cell migration alone, but the inclusion of the sequence encoded by the alternative last exon, rather than the RGG domain, contributes to the robust phenotype observed in the ntEWS expressing cells.

### Androgen signaling promotes *EWSR1* breakpoint formation via R-loops

**Figure 5.**
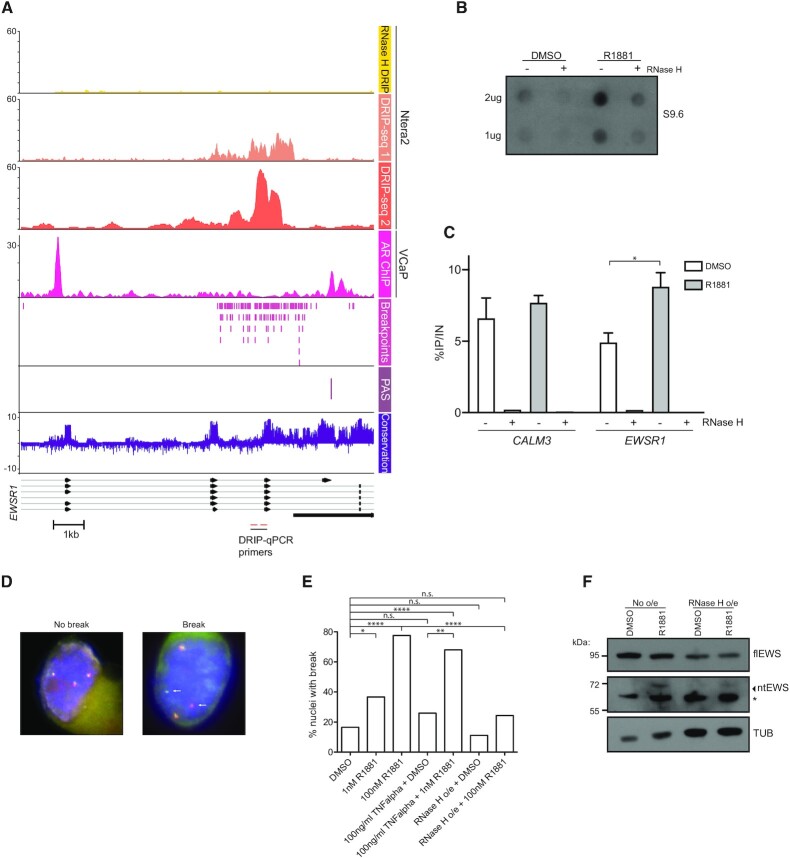
Androgen signaling promotes *EWSR1* breakpoint formation via R-loops. (**A**) DRIP-seq enrichment from (56) shown from exon 6 to exon 10 of *EWSR1*. Breakpoint coordinates in *EWSR1* are from the Catalog of Somatic Mutations In Cancer (COSMIC) (66). Conservation is from multiple alignment of 100 vertebrate species. (**B**) Dot blot using DNA/RNA hybrid antibody for the labeled conditions. (**C**) DRIP-qPCR for *CALM3* and intron 5 of *EWSR1* from VCaP cells treated with DMSO or 100 nM R1881 for 24 h. RNase H treatment as indicated. Shown is percent IP/Input as mean ± SEM for at least three replicates (*P <0.05). RNase H treated IPs were performed once. (**D**) Representative images of nuclei with *EWSR1* alleles intact (No break) or split (Break). White arrows indicate a break apart allele. (**E**) Quantification of percent nuclei with a break. VCaP cells were treated with indicated doses of R1881 for 24 h. TNFα treatment was for 48 h. *P* values (*P < 0.05, **P < 0.001, ****P < 0.0001) were calculated using chi-square (*N* = 85, 30, 67, 27, 25, 36, 37). (**F**) Immunoblot of flEWS, ntEWS, and tubulin (TUB) in designated conditions after treatment with DMSO or 100 nM R1881 for 24 h. Differences in protein loading were not intentional, but further emphasize the loss of ntEWS when RNase H is present. See also Supplementary Figure S5.

The *ntEWS* PAS is in close genomic proximity to the sequence in *EWSR1* that recurrently rearranges with *FLI1* in Ewing sarcoma, the breakpoint hotspot. Therefore, we hypothesized that in addition to regulating *ntEWS* expression, AR could regulate *EWSR1* breakpoint formation. In fact, plotting the AR ChIP-seq dataset from VCaP cells shows AR binding flanks the breakpoint hotspot (Figure 5A). This was particularly curious since AR binding to *TMPRSS2* and *ERG* introns promotes formation of the *TMPRSS2/ERG* gene rearrangement in prostate cancer (25). Further, another nuclear hormone receptor, Estrogen Receptor, promotes breast cancer associated translocations by stimulating R-loop formation at target genes (52). Therefore, we investigated the importance of R-loops in *EWSR1* breakpoint formation as these can be a hotspot for DNA damage (53). Analysis of R-loops by DNA:RNA immunoprecipitation followed by next-generation sequencing (DRIP-seq) in a cell line of embryonic carcinoma of the testis (NTERA2) (38) showed that the same sequence in *EWSR1* that harbors the breakpoint hotspot forms an R-loop (Figure 5A). The breakpoint R-loop is resolved by treatment with RNase H, which degrades the R-loop-associated RNA. To determine if androgen signaling impacts R-loop abundance genome wide, R-loop abundance was measured in VCaP cells treated with DMSO or 100 nM R1881 for 24 h by dot blot using the S9.6 DNA:RNA hybrid antibody (Figure 5B and Supplementary Figure S5A). Treatment with R1881 caused an increase in R-loops genome wide and this signal was diminished by RNase H (Figure 5B). DRIP-qPCR showed an R1881-dependent increase in R-loops at the *EWSR1* locus, but an R1881 independent R-loop at the *CALM3* gene, which is a known R-loop hotspot (Figure 5C).

To test the role of androgen signaling in *EWSR1* breakpoint formation, a break apart fluorescence *in situ* hybridization assay (FISH) was used. Fluorescent probes flank the breakpoint hotspot and create a merged red and green signal (yellow) when the *EWSR1* gene is intact and split signal when a break at breakpoint hotspot has occurred (Figure 5D). Treatment of VCaP cells with R1881 caused increased break formation in a dose dependent manner (Figure 5E), with a striking 80% of cells showing *EWSR1* breakage at the supraphysiological dose of 100 nM. Because high levels of androgen can cause DNA damage (54), we asked if lower levels of androgen signaling could also promote high frequency breakage of *EWSR1*. Mani *et al.* reports that inflammation induced oxidative stress mediated by TNFα combined with androgen signaling promotes formation of the TMPRSS2/ERG fusion in prostate cells (55). We found that VCaP cells treated with TNFα alone did not show significantly increased *EWSR1* breakpoint frequency, however the combination of low dose (1 nM) R1881 with TNFα increased breakpoint frequency to near that of cells treated with high dose (100 nM) R1881 (Figure 5E). These data suggest that androgen signaling can produce the fusion forming break at *EWSR1* alone at high doses or at low doses in collaboration with inflammation induced stress. To test the importance of R-loops in *EWSR1* breakpoint formation, RNase H was expressed in VCaP cells, treated with DMSO or 100 nM R1881 (Supplementary Figure S5B), and these cells were assayed by break apart FISH. RNase H significantly abrogated breakpoint formation in cells with 100 nM R1881 (Figure 5E). This suggests that R-loops are essential for androgen-mediated *EWSR1* breakpoint formation.

Transcripts commonly terminate through the formation of transcription dependent R-loops (56). RNase H expression in VCaP cells abolished expression of ntEWS and decreased expression of flEWS (Figure 5F). This is consistent with both 5′ and 3′ R-loops in the EWSR1 gene (Supplementary Figure S5C). These data suggest that R-loops can regulate both flEWS and ntEWS expression and can promote break-point formation.

## DISCUSSION

We have shown in the prostate cancer setting that AR binds to intron 5 of *EWSR1* to directly upregulate a previously uncharacterized isoform that we have termed ntEWS. Our data indicate that ntEWS is localized to the cytoplasm and can strongly promote phenotypes associated with cancer such as cell migration and clonogenic growth. Further, AR signaling promoted increased R-loop formation in the EWSR1 gene and drove chromosomal breakage at high frequency at the same genomic locus that is rearranged in Ewing sarcoma.

Alternative RNA processing events can give rise to tissue specific gene isoforms (43), and the misexpression of such isoforms is common in cancer (57). Specifically, mRNA shortening through alternative polyadenylation is common in cancer (58). Yet exactly how RNA processing becomes dysregulated, what factors dictate this process in *cis* and in *trans*, and the downstream cellular consequences remain unclear. While 43.5% of AR binding sites are intronic (59), the exact role of AR-bound introns is under-examined and is likely context specific. Interestingly, AR intronic binding to the *TSC2* gene is associated with expression of a truncated cytoplasmic isoform of TSC2 that increases cell proliferation (60,61). In the context of *EWSR1*, our data indicate that intronic AR binding stimulates early termination. These data suggest that AR functions to regulate isoform expression, however the exact mechanism is still unclear. RNA processing is inherently coupled to transcription, and many transcription factors, including AR, interact with RNA processing factors. For example, the RNA processing factors NONO and SFPQ interact with AR to increase transcriptional output (62–64). Future work is needed to test if AR can alter RNA processing via these or other factors.

We found that *ntEWS* expression is normally high in the testes. Because this organ is androgen regulated, this may explain why *ntEWS* expression is under control of AR. Mouse knockout models for *EWSR1* and *AR* are both defective in spermatogenesis due to meiotic arrest and increased apoptosis (1,26), indicating a potential relationship between these genes in the testes. It is important to note that the Cre-Flox targeting strategy used in these studies to delete *EWSR1*, did eliminate essential exons for *ntEWS* (1). Our data indicate that ntEWS may function in the cytoplasm and can form the higher order structure associated with phase separation. Interestingly, in full-length EWS, RNA can titrate the formation of higher order structures through binding the EWS RNA binding domain (19). Since ntEWS lacks this RNA binding domain, higher-order structures formed by ntEWS would not be regulated by this mechanism.

Unlike many other gene fusions, we do not have a mechanistic explanation for why *EWSR1/FLI1* forms. Ewing sarcoma preferentially affects adolescent males with peak incidence at 15 years of age (4). This affected population is typically undergoing puberty, a period of peak androgen signaling. Our data suggest that this increase in androgen signaling, possibly combined with inflammatory signaling could promote the chromosomal breaks necessary for *EWSR1/FLI1* formation. The origins of the most common fusion in prostate cancer, *TMPRSS2/ERG* is accredited to intronic AR binding in combination with DNA damage or inflammatory signaling (25,55). Our current study suggests a similar mechanism could lead to to *EWSR1* breakpoint formation followed by *EWSR1/FLI1* fusion. Since the *EWSR1* breakpoint hotspot is so close to the intronic PAS, we speculate that alternative polyadenylation stress may be related to breakpoint formation. Although we were able to generate *EWSR1* breakpoints at high frequencies in a prostate cancer cell line, *EWSR1* rearrangements are not common in prostate cancer. A recent study did find a *EWSR1/FEV* fusion in a prostate tumor from a single patient, but it is unclear whether this tumor should be categorized as prostate adenocarcinoma or Ewing sarcoma (65). We hypothesize that EWS/FLI1 fusions are unlikely in prostate cells because *FLI1* is not expressed and is likely in heterchromatin. Our data indicate that high levels of AR in the cell are necessary for AR binding and function within the *EWSR1* gene. It is unclear if the cell of origin for Ewing sarcoma would express AR. While mesenchymal stem cells are a likely Ewing sarcoma cell of origin, the exact subset of these cells and their state when *EWSR1/FLI1* forms is unknown. Future studies that stimulate both AR and inflammation in a mesenchymal cell setting are needed to test this potential mechanism for gene rearrangement.

## DATA AVAILABILITY

Quantitative PCR complies with MIQE guidelines. See Supplementary Tables S1 and S2.

## Supplementary Material

zcab033_Supplemental_File
