# Supplementary material for: Androgen signaling connects short isoform production to breakpoint formation at Ewing sarcoma breakpoint region 1
Source: NAR Cancer. 2021 Aug 14;3(3):zcab033. doi: 10.1093/narcan/zcab033 (PMC8364332; doi:10.1093/narcan/zcab033)
Supplement: zcab033_Supplemental_File [file zcab033_supplemental_file.pdf]

A

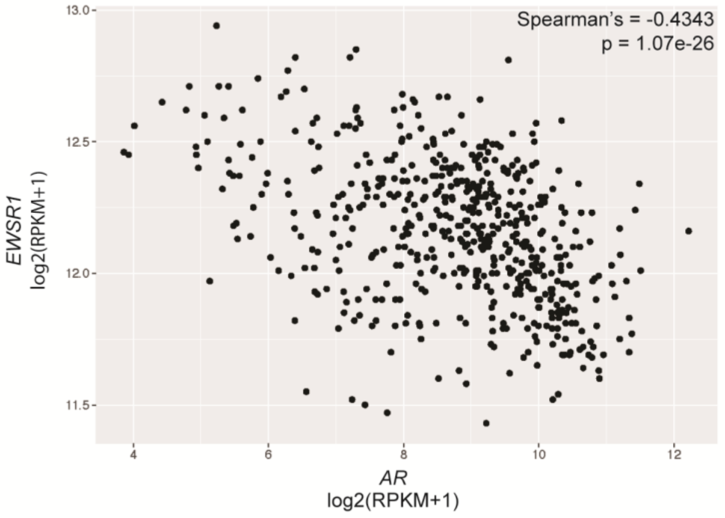

B

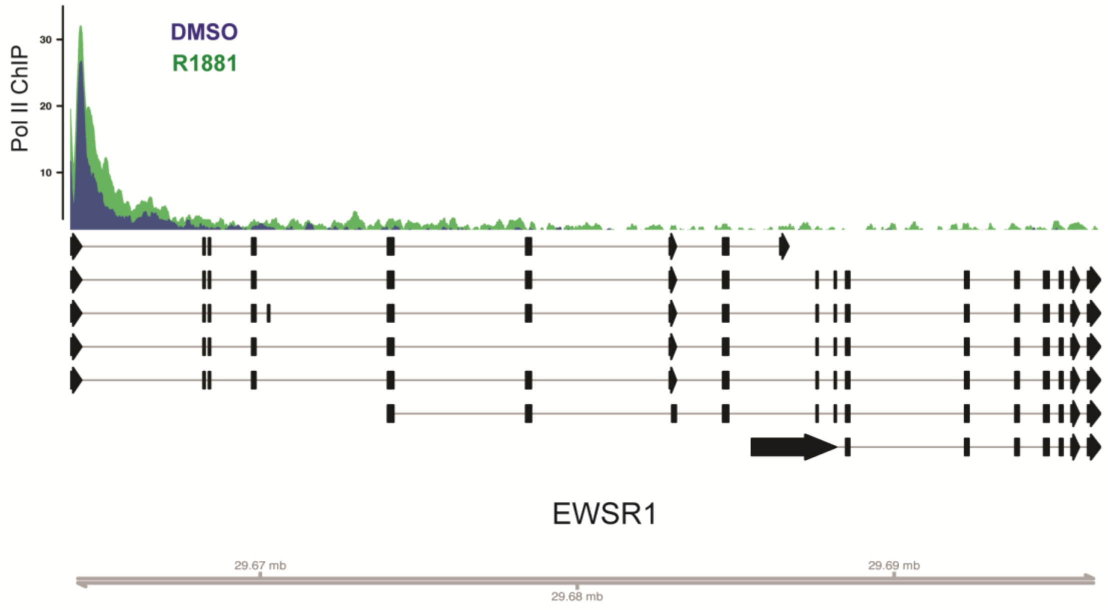

Figure S1. Androgen signaling upregulates an intronic polyadenylated *EWSR1* isoform A. Gene expression correlation of *EWSR1* and *AR* in 550 prostate cancer patients from the PRAD data set. **B.** Pol II ChIP-seq after treatment with DMSO or R1881 at *EWSR1* in VCaP cells. Data analyzed from Baumgart et al. Mol Oncol 2020.

A

	% of samples with expression	PAS signal	Mean RPM	Location
1	4.7	AAUAAA	0.8	Intron
2	69.2	AUUAAA	3.0	Intron
3	2.8	None	1.4	Intron
4	1.9	None	1.8	Coding exon
5	77.6	AUUAAA	3.5	3' exon
6	57.9	None	3.4	3' exon
7	55.1	AAUAAA	3.0	3' exon

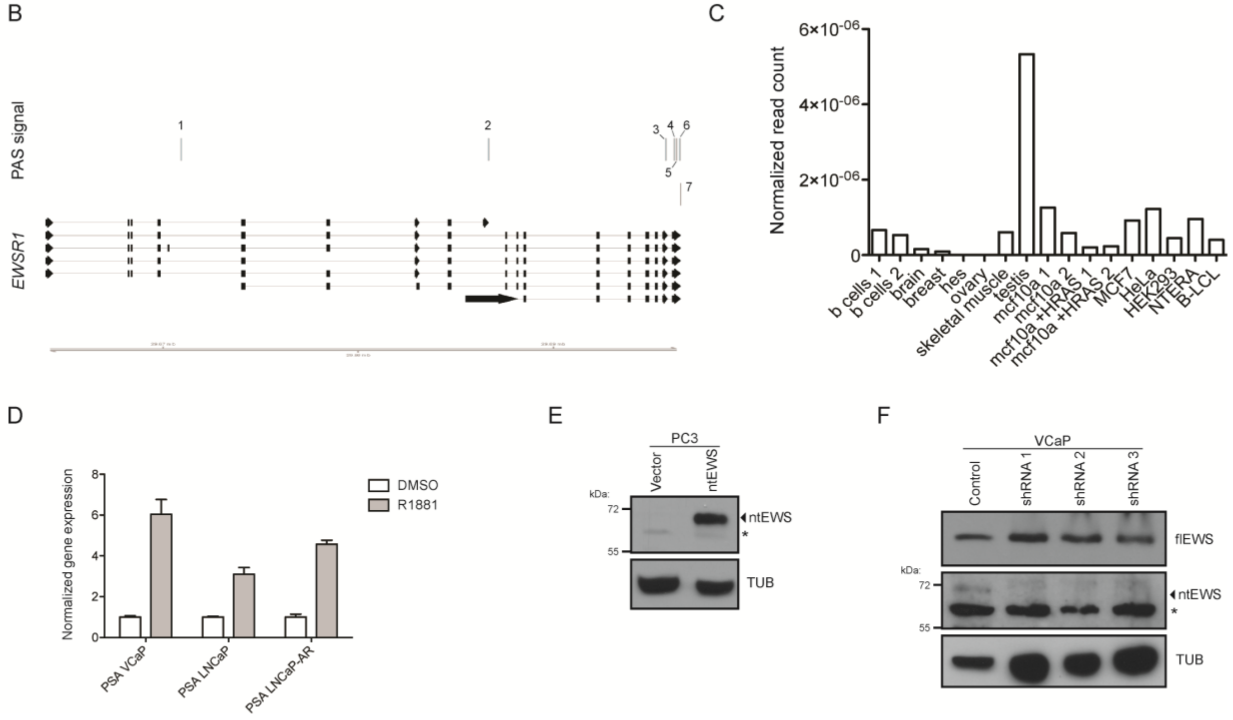

Figure S2. AR binding to Intron 5 of *EWSR1* directly regulates ntEWS expression A. *EWSR1* polyadenylation site (PAS) information from PolyA_db v3.2. There are seven PAS for this gene and they are numbered starting from the 5' end of the gene. **B.** Diagram of PAS locations at *EWSR1* numbered according to table in A. **C.** Normalized read count for PAS #2, the PAS for *ntEWS*, from 3' sequencing data across various cell types. **D.** RNA levels of PSA in VCaP, LNCaP, and LNCaP-AR cells treated with DMSO or 10nM R1881 for 24 hours. Expression is normalized to 18S and relative to the DMSO condition. The mean ± SEM for three replicates is shown. **E.** Immuno blot of ntEWS in PC3 cells overexpressing HA-ntEWS. **F.** Immunoblot of ntEWS in VCaP cells overexpressing vector alone or shRNAs targeting the 3' UTR of ntEWS. Tubulin is used as a loading control for immunoblots.

A

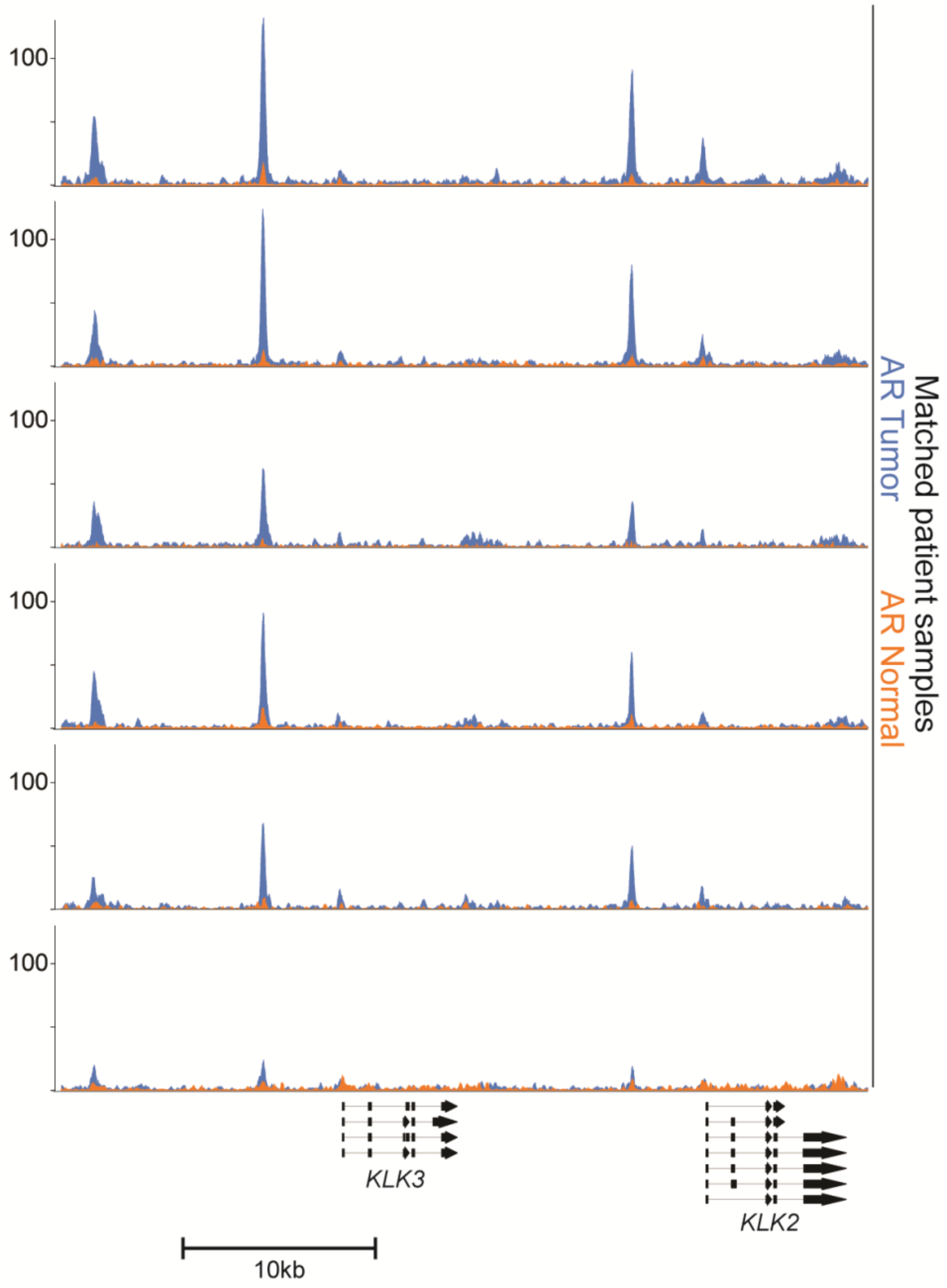

Figure S3. Gene tracks for AR binding in patient tumor and matched adjacent normal tissue at known AR enhancers. Order of tracks is consistent with Figure 2a. Data analyzed from Pomerantz et al. Nat Genet 2015, Toropainen et al. NAR 2015, and Malinen et al. NAR 2017.

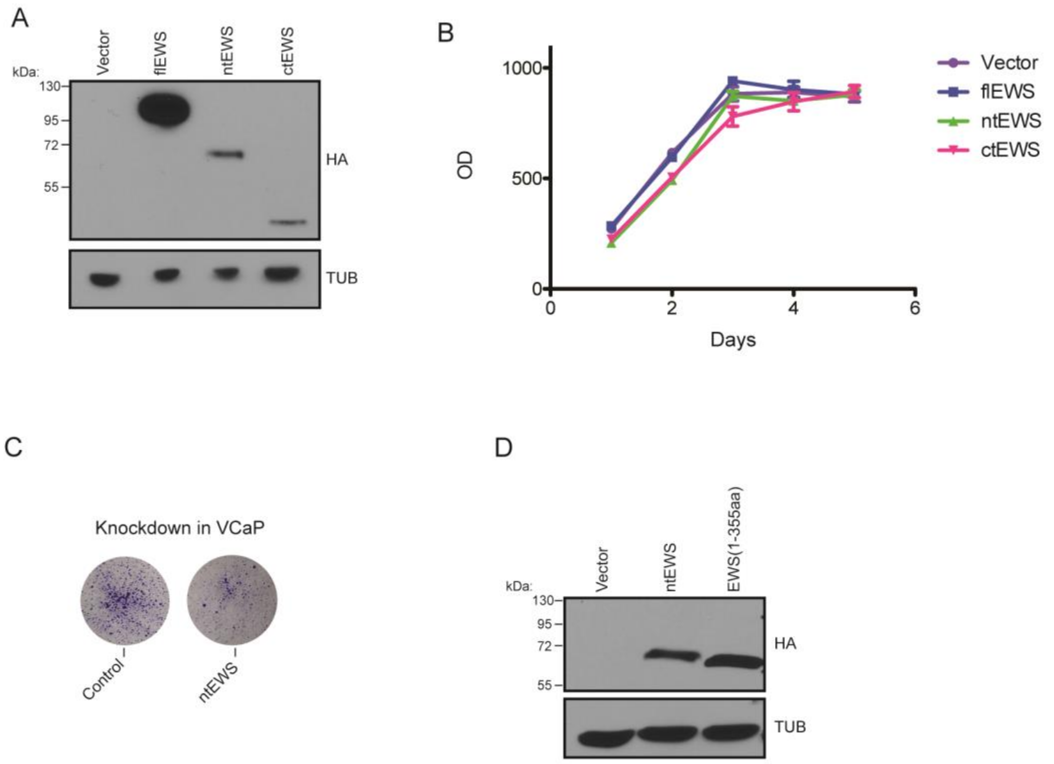

Figure S4. ntEWS promotes phenotypes related to oncogenesis A. Immunoblot of 3xHA tagged EWS isoforms expressed in PC3 cells. Tubulin is used as a loading control. **B.** MTT proliferation assay of PC3 isoform-expressing lines. **C.** Representative images of clonogenic growth assay quantified in Figure 4E. **D.** Immunoblot of 3xHA tagged ntEWS and EWS (1-355aa) expressed in PC3 cells in addition to empty vector. Tubulin is used as a loading control.

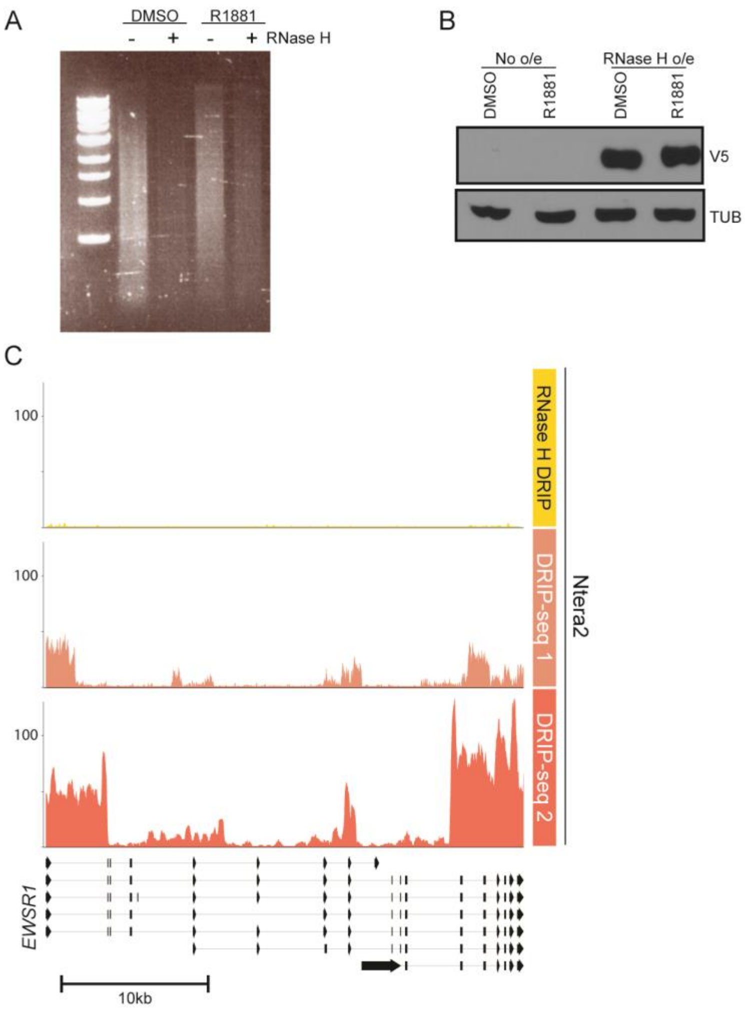

Figure S5. Androgen signaling promotes *EWSR1* breakpoint formation via R-loops A. Agarose gel image of DRIP input from VCaP cells. **B.** Immunoblot for V5-tagged RNase H in VCaP cells either treated with DMSO or 10nM R1881. Tubulin is used as a loading control. **C.** DRIP-seq tracks at *EWSR1* as labeled. Data analyzed from Sanz et al. Nat Protoc 2019.

Supplementary Table S1. qPCR Target Data

Target	Sequence	Amplicon Size	Primer Location	Splice Variants Targeted	Target Sequence Accession Number
flEWS forward	TTATGGGCAGGAGTCTGGAGG	203 bp	Exon 8	Variants 1-4	NM_001163285
flEWS reverse	CTGGTCCTTCATCCATGGGTC	203 bp	Exon 10	Variants1-4	NM_001163285
ntEWS forward 1	GGAGGATTTTCCGGACCAGG	153 bp	Exon 8	Variant 5	NM_001163287
ntEWS reverse 1	GTATACAAGGCTCTCACTTTG	153 bp	Exon 9	Variant 5	NM_001163287
ntEWS forward 2	GGAGGATTTTCCGGACCAGG	219 bp	Exon 8	Variant 5	NM_001163287
ntEWS reverse 2	CTAGTCCCACTTTTCATTATGC	219 bp	Exon 9	Variant 5	NM_001163287
PSA forward	GTGACCAAGTTCATGCTGTG	206 bp	Exon 4	Variant 1,3,4	NM_001648
PSA reverse	TTGGCCACGATGGTGTCCTTG	206 bp	Exon 5	Variant 1,3,4	NM_001648
18s forward	GGTGAAATTCTTGGACCGGC	196 bp	Exon 1	-	NR_145820.1
18s reverse	GACTTTGGTTTCCCGGAAGC	196 bp	Exon 1	-	NR_145820.1
EWSR1 intron 5 forward	GCGTTTACTGTGATGAATGGAGC	214 bp	Intron 5	-	-
EWSR1 intron 5 reverse	CTCCTGGGTAAGAATGCTAC	214 bp	Intron 5	-	-
EWSR1 intron 8 forward	TGCATGCAACAGCTTGAAAT	155 bp	Intron 8	-	-
EWSR1 intron 8 reverse	GAGGGGAGAGGGAAATATGAA	155 bp	Intron 8	-	-
XKRT forward	GGGATGGAGGTTTGCTCTTG	145 bp	Intron 3	-	-
XKRT reverse	TGGACATGGTAGCGGGTAC	145 bp	Intron 3	-	-
CALM3 DRIP forward	GAGGAATTGTGGCGTTGACT	169 bp	Exon 6	-	-
CALM3 DRIP reverse	AGAGTGGCCAAATGAGCAGT	169 bp	Exon 6	-	-
EWSR1 DRIP forward	CCTTGGTTAGTGCCTTGGAA	119 bp	Intron 8	-	-
EWSR1 DRIP reverse	GTCGGAATGAACCTGAGGAA	119 bp	Exon 9	-	-

Supplementary Table S2. qPCR standard curve data

Figure 1E

Target	18S	flEWS	ntEWS1	ntEWS2
Slope	-3.441	-3.613	-3.310	-3.386
Y-intercept	38.06	38.17	34.20	34.12
Efficiency	0.95	0.89	1.00	0.97
R^2	0.993	0.993	0.998	0.996
LDR	10^{10}-10^{7}	10^{8}-10^{4}	10^{6}-10^{2}	10^{6}-10^{2}

Figure 1F

Target	18S	flEWS	ntEWS1	ntEWS2
Slope	-3.98	-3.702	-3.366	-3.326
Y-intercept	40.37	40.01	36.86	36.24
Efficiency	0.86	0.86	0.98	1.00
R^2	0.993	0.993	0.998	0.998
LDR	10^{10}-10^{7}	10^{8}-10^{4}	10^{6}-10^{2}	10^{6}-10^{2}

Figure S1E

Target	18S VCaP	18S LNCaP	18S LNCaP AR
Slope	-3.441	-3.698	-4.462
Y-intercept	38.06	40.37	47.818
Efficiency	0.95	0.86	0.675
R^2	0.993	0.993	0.986
LDR	10^{10}-10^{7}	10^{10}-10^{7}	10^{10}-10^{7}

Figure S1E

Target	PSA VCaP	PSA LNCaP	PSA LNCaP AR
Slope	-4.018	-4.013	-3.523
Y-intercept	38.92	41.30	39.296
Efficiency	0.77	0.77	0.92
R^2	0.991	0.995	0.945
LDR	10^{8}-10^{5}	10^{8}-10^{5}	10^{8}-10^{5}

Figure 2E

Target	18S	flEWS	ntEWS1
Slope	-4.403	-4.147	-3.406
Y-intercept	46.414	46.414	36.005
Efficiency	0.678	0.74	0.966
R^2	0.992	0.996	0.968
LDR	10^{10}-10^{7}	10^{8}-10^{4}	10^{6}-10^{2}

Supplementary Table S3. Cloning and gRNA Primers

	5’	3’
**Cloning primers**		
HA-ntEWS	CTATGCATACCCATACGATGTTCCAGATTACGCTAAGGCGTCCACGGATTACAGTACCTAT	GTGACATTAATTAACTAGTCCCACTTTTCATTATGCTGCCG
HA-ctEWS	CTATGCATACCCATACGATGTTCCAGATTACGCTAAGGATGAAGGACCAGATCTTGAT	GTGACATTAATTAATTACTAGTAGGGCCGATCTCTGCG
HA-EWS (1-355aa)	CTATGCATACCCATACGATGTTCCAGATTACGCTAAGGCGTCCACGGATTACAGTACCTAT	GTGACATTAATTAATTACTAGTAGGGCCGATCTCTGCG
3xHA	AGACTGCGGCCGCATGTATCCGTATGACGTCCCGGACTATGCATATCCGTATGACGTCCCGGACTATGCATACCCATACGATGTTC	
AR	AGACTGCGGCCGCATGGAAGTGCAGTTAGGGCTG	GTGACATTAATTAATCACTGGGTGTGGAAATAGAT
**gRNA primers**		
Downstream FOXA1:AR site	CACCGAGCTTTGTAGCATTCTTACCC	AAACGGGTAAGAATGCTACAAAGCTC
Upstream FOXA1:AR site	CACCGATCCGGGAGAAGTGATCTGTT	AAACAACAGATCACTTCTCCCGGATC
